# Single-stage surgical repair of a pre-coarctation aortic arch aneurysm and arteria lusoria

**DOI:** 10.1093/icvts/ivae220

**Published:** 2024-12-30

**Authors:** Mouhammad Kanj, Ziad Mansour, Fadi Farhat

**Affiliations:** Cardiothoracic Surgery Department, Lebanese Geitaoui University Medical Centre, Beirut, Lebanon; Cardiothoracic Surgery Department, Lebanese Geitaoui University Medical Centre, Beirut, Lebanon; Cardiovascular Surgery Department, Alain Sisteron Institute, Infirmerie Protestante de Lyon, Caluire-et-Cuire, France

**Keywords:** Coarctation, Aneurysm, Arteria lusoria, Surgical repair

## Abstract

Managing an adult patient with aortic coarctation and associated anomalies presents a significant surgical challenge. We present a case of an adult male with aortic coarctation, pre-coarctation distal arch 7-cm aneurysm involving the origin of the left subclavian artery, and aberrant (lusoria) right subclavian artery. He was managed with one surgical approach, consisting of right carotid-subclavian bypass, exclusion of the right subclavian artery, proximal descending aortic replacement and reinsertion of left subclavian artery, using partial cardiopulmonary bypass.

## INTRODUCTION

Coarctation of the aorta is diagnosed in childhood, accounting for 4% of congenital heart diseases [1]. However, in up to 20% of cases, coarctation may go unnoticed. Ninety percent of surviving untreated patients will die before 55 [2]. Aneurysm formation around the coarctation is a complication of untreated coarctation. We present the case of an adult with a previous surgical ventricular septal defect (VSD) closure in childhood and who was diagnosed with aortic coarctation with pre-coarctation saccular aneurysm involving the left subclavian artery and an aberrant (lusoria) right subclavian artery. We detail the surgical strategy for avoiding redo sternotomy and subsequent cardiac arrest.

## CASE PRESENTATION

A 38-year-old man with medical record of VSD closure at the age of 6 with residual high pulmonary hypertension treated with macitentan, stable over the past 10 years (New York Heart Association II) was admitted for chest pain.

**Figure 1:**
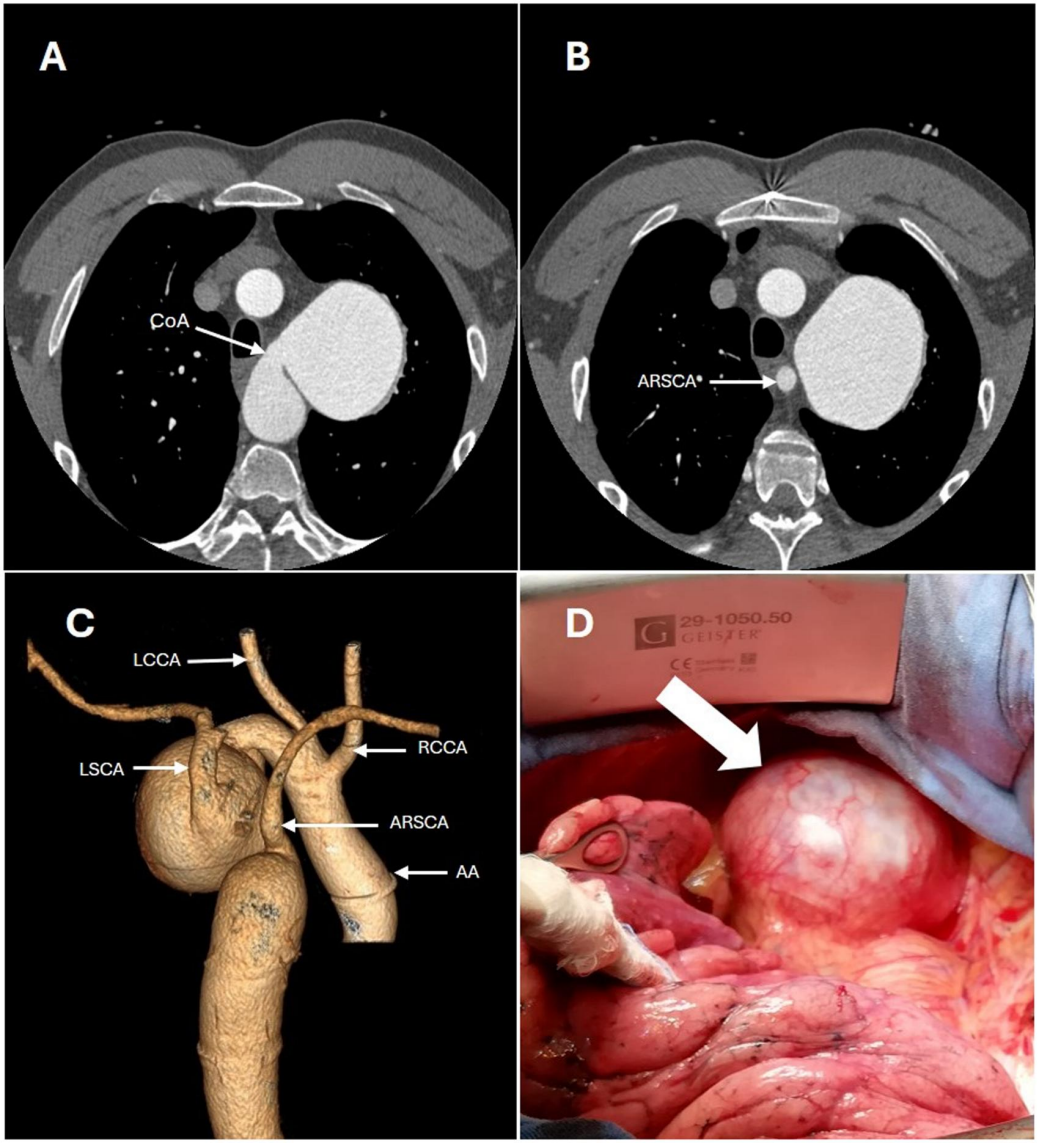
(**A**) Preoperative CT scan. (**B**) Pre-coarctation aneurysm with the arteria lusoria. (**C**) 3D reconstruction (the aorta is rotated 180°). (**D**) Intraoperative view of the pre-coarctation aneurysm through a left thoracotomy. AA: ascending aorta; ARSCA: aberrant right subclavian artery; CoA: coarctation of the aorta; LSCA: left subclavian artery; RSCA: right subclavian artery; Thick arrow: saccular aneurysm.

Echocardiography and computed tomography (CT) angiography confirmed a bicuspid aortic valve (BAV) and coarctation of the aorta. Proximal to the coarctation was a 7-cm saccular aneurysm involving the origin of the left subclavian artery. Distal to the coarctation, an aberrant right subclavian artery was identified (Fig. 1A–C).

Echocardiography showed a preserved right ventricle with a systolic pulmonary artery pressure (75 mmHg). The left ventricle and the BAV exhibited normal functions. Due to the discrepancy in between the distal arch and the descending aortic diameters, as well as the presence of an abnormal right subclavian artery, we excluded an endovascular management, opting for a surgical repair using a double-stage approach. Because of the pulmonary hypertension, we excluded an approach through redo sternotomy to avoid cardiac arrest and subsequent right ventricular disfunction.

## OPERATIVE TECHNIQUE

Anaesthetic equipment included a double lung ventilation and a left jugular catheter. Two arterial catheters (right radial and right femoral) were inserted to assure the blood pressure monitoring. The procedure was performed under normothermia. First, a right carotid-subclavian bypass using an 8-mm Dacron graft was performed in a supine position. The patient was then moved to right lateral decubitus allowing a left thoracotomy in the 5th intercostal space. The proximal descending aorta and the left subclavian artery were freed. The arteria lusoria was ligated at arousal. The aortic arch was dissected (Fig. 1D).

A partial cardiopulmonary bypass (CPB) was initiated with a flow rate of 2 l/min between the pulmonary artery (inflow) and the distal descending aorta (outflow). The aorta was clamped after the left common carotid artery and below the aneurysm. The left subclavian artery was clamped and cut. The aneurysm and coarctation were removed and replaced by a 28-mm Dacron graft. The descending aorta was declamped after 10 min and the CPB weaned after 19 min.

**Figure 2:**
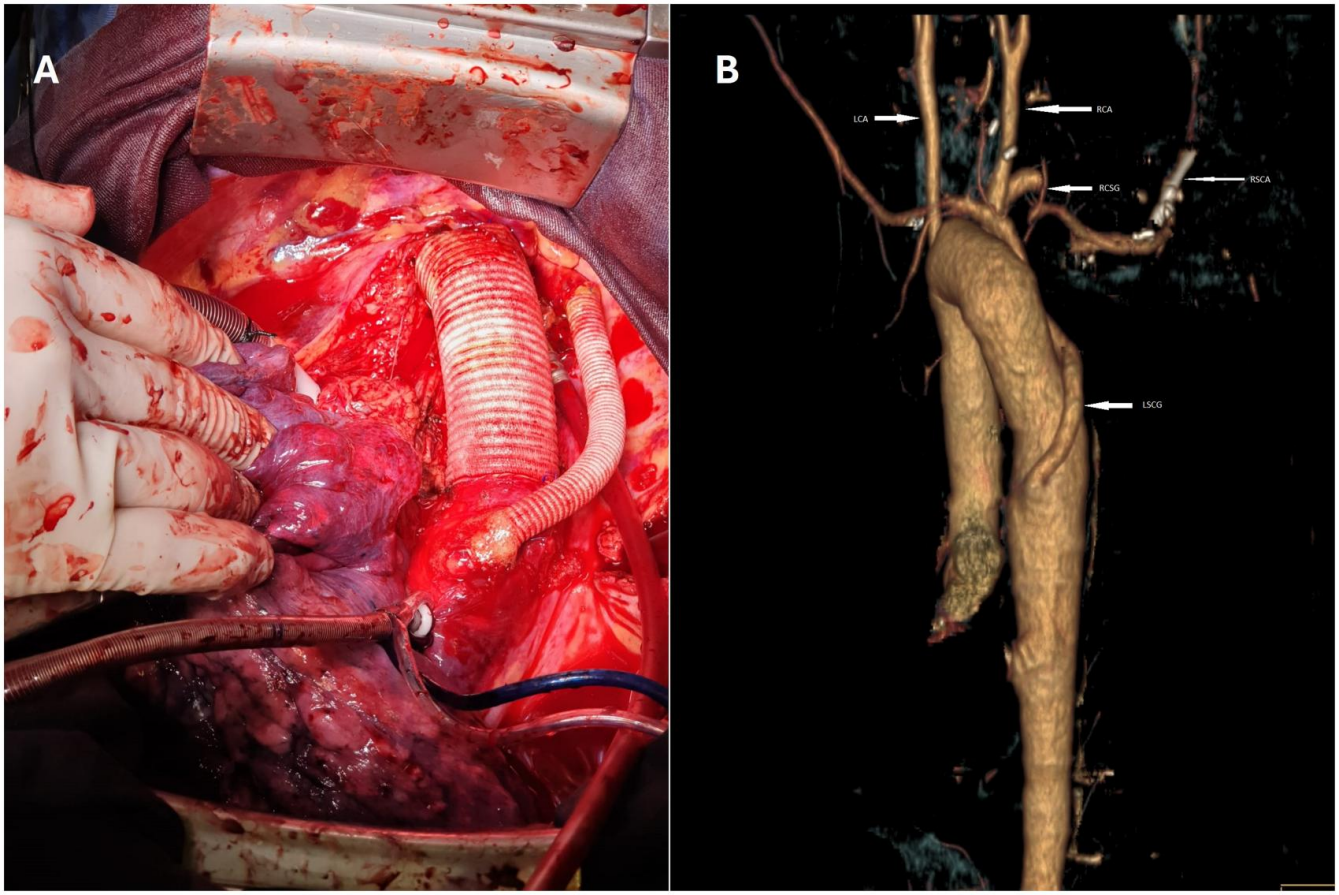
(**A**) Intraoperative view of the final result. (**B**) 3D reconstruction of CT angiography 1-year post-surgery. LCA: left carotid artery; LSCG: left subclavian artery graft; RCA: right carotid artery; RSCA: right subclavian artery; RCSG: right carotid subclavian graft.

An 8-mm Dacron graft was anastomosed between the left subclavian artery and the descending aorta below the level of the Dacron tube proximally under lateral clamping of the aorta (Fig. 2A) before protamine, decannulation and chest closing.

The patient was transferred to the ward on day 2 and discharged home on day 12. He fully recovered after 4 weeks. The 1-year follow-up CT angiography was normal (Fig. 2B). Last follow-up was performed after 2 years, without clinical modification.

## DISCUSSION

Coarctation of the aorta is a congenital heart condition sometimes diagnosed in adulthood, often during assessments of systemic hypertension. In patients with aortic coarctation, approximately 50% to 80% have a BAV, which is associated with aortic medial degeneration and aneurysm formation [3]. Warnes characterized coarctation as a diffuse arteriopathy and part of the disease spectrum linked to BAV [1]. If left untreated, coarctation can lead to aneurysms on the aortic arch, significantly raising the risk of rupture and death [4].

The combination of arteria lusoria and coarctation is uncommon [5]. Historical data include Abbott (2/300 cases), Broden (3/150 cases) and Richter (5/137 cases). Arteria lusoria can originate either proximal or distal to the coarctation [5].

Strategies for patients with complex aortic diseases vary widely. Endovascular approaches are used for cases where the anatomy of the arch is suitable. In our patient, a pre-coarctation aneurysm was present alongside an arteria lusoria located just beyond the coarctation. Managing aneurysms in patients with coarctation is challenging due to the elevated risk of rupture and the discrepancy in between the different aortic segments. Consequently, we have decided to exclude an endovascular approach.

Due to the involvement of the left subclavian artery with the aneurysm and the presence of an arteria lusoria, both vessels were resected at arousal before revascularization. A double-stage repair was decided, beginning with a right carotid-subclavian bypass, then proceeding with a left thoracotomy for descending aortic replacement under partial CPB and reinsertion of the left subclavian artery. This approach seemed less complex than a midline redo sternotomy with circulatory arrest for arch replacement. Reimplantation of the left subclavian Dacron tube graft on the descending aorta was performed after the weaning of CPB to shorten the time of bypass.

## CONCLUSION

In this patient, our approach allowed a complete management of the aortic disease with an acceptable surgical risk, especially by eliminating a cardioplegic arrest knowing the severe pulmonary hypertension.

**Conflict of interest:** None declared.

## Data Availability

The data that support the findings of this study are available from the corresponding author upon reasonable request.
